# Spectroscopic Nuclear Magnetic Resonance and Fourier Transform–Infrared Approach Used for the Evaluation of Healing After Surgical Interventions for Patients with Colorectal Cancer: A Pilot Study

**DOI:** 10.3390/cancers17050887

**Published:** 2025-03-05

**Authors:** Lavinia Raluca Șaitiș, David Andras, Ioana-Alina Pop, Cătălin Șaitiș, Ramona Crainic, Radu Fechete

**Affiliations:** 1 Doctoral School, Faculty of Physics, Babeş-Bolyai University, 1 Kogălniceanu, 400084 Cluj-Napoca, Romania or laviniadragann@yahoo.com (L.R.Ș.); or ramona.crainic95@gmail.com (R.C.); 2 Faculty of Material and Environmental Engineering, Technical University of Cluj-Napoca, 103-105 Muncii Bulevard, 400641 Cluj-Napoca, Romania; 3 Surgical Department, County Emergency Hospital, Clinicilor Str. 3-5, 400009 Cluj-Napoca, Romania; dr.andras@gmail.com; 4 Surgical Department, Faculty of General Medicine, Iuliu Hațieganu University of Medicine and Pharmacy, Victor Babeș Str. 8, 400012 Cluj-Napoca, Romania; 5 Radiology Department, County Emergency Hospital, Clinicilor Str. 3-5, 400009 Cluj-Napoca, Romania; ioanapop99@yahoo.com; 6 Faculty of Construction, Technical University of Cluj-Napoca, 25 Barițiu, 400641 Cluj-Napoca, Romania; saitis@mail.utcluj.ro

**Keywords:** colorectal cancer, evolution of healing process after surgery, ^1^H NMR relaxometry, FT-IR spectroscopy, PCA analysis, ROC curves and AUC, Prediction using artificial intelligence

## Abstract

**Background/Objectives:** Colorectal cancer (CRC) is one of the most common and deadly types of cancer. Compared with the classical histopathological approach, this study discusses the application of ^1^H NMR and FT-IR techniques for the fast evaluation degree of healing of patients with CRC after surgical intervention. **Methods:** Native and deproteinized blood plasma collected from 10 patients with confirmed CRC and 20 healthy volunteers were analyzed using ^1^H NMR *T*_2_ distributions and FT-IR spectra measured for samples collected before and 7 days after surgery. The average FT-IR spectrum from 20 healthy volunteers is also presented. Principal component analysis (PCA) was performed on the FT-IR spectra. The results were used for further statistical analysis using receiver operating characteristic (ROC) and area under the curve (AUC) and to produce a series of prediction maps using a machine learning library. **Results:** Both experimental methods combined with analysis methods demonstrated that the native blood plasma samples can be better used to predict the CRC patients’ evolution 7 days after surgery. Three patients showed a significant evolution by ^1^H NMR *T*_2_ distribution, correlated to the observation of FT-IR–PCA analysis. Maps of medical state probability were generated using a trained machine learning-based ANN. **Conclusions:** The experimental measurements combined with an advanced statistical analysis and machine learning were successfully used and show that the healing process of patients with CRC is not linear, from the preoperative state to the state associated with healthy volunteers, but passes through a distinct healing state

## Simple Summary

Native and deproteinized blood plasma collected from 10 patients with confirmed CRC, before and 7 days after surgery, and from 20 healthy volunteers were measured by ^1^H NMR *T*_2_ relaxometry and FT-IR spectroscopy and statistically analyzed by PCA, ROC and AUC and by prediction maps using machine learning-based ANN. ^1^H NMR relaxometry and FT-IR spectroscopy methods combined with numeric analysis methods demonstrated that the native blood plasma samples can be better used to predict the evolution of patients with colorectal cancer at 7 days after surgery. Successful individual and group evolutions were discussed and a nonlinear healing evolution was observed and evaluated.

## 1. Introduction

Colorectal cancer (CRC) is the third most frequently diagnosed malignancy and the third leading cause of cancer-associated mortality in the United States [1]. In 2020, colorectal cancer ranked as the third most frequently diagnosed type of cancer worldwide, with approximately 1.9 million new cases and causing 930,000 deaths. Epidemiological projections indicate that by 2040, the incidence of this type of cancer will increase significantly, reaching 3.2 million new cases annually, while the associated mortality is expected to rise to 1.6 million deaths per year [2]. It holds the second position in overall cancer-related deaths and is the primary contributor to cancer-related fatalities in males below the age of 50. More than half of all CRC cases can be attributed to modifiable risk factors, such as tobacco use, an unhealthy dietary pattern, excessive alcohol intake, lack of physical activity, and obesity [1]. A substantial number of CRC cases and mortality can be averted through regular screening, vigilant monitoring and access to high-quality medical care [3].

Serum markers such as carcinoembryonic antigen (CEA) and cancer antigen 19-9 (CA 19-9) have low specificity and sensitivity [4]. Colonoscopy, one of the most widely available diagnostic methods, has a high degree of invasiveness [5]. Nowadays, using specific tumor markers and imaging methods, oncology patient management is based on tumor progression determined by tumor size, the degree of lymphatic dissemination, the number of affected lymph nodes, the presence of distant metastases and other comorbidities [6,7]. Computed tomography colonography (virtual colonoscopy or colono-CT), a fast and non-invasive investigation, is a promising alternative. However, when anomalies are detected or depending on the size of the lesions, it has reduced performance in detecting lesions smaller than 5 mm or flat lesions, which, although rare, may have malignant potential. In such cases, conventional colonoscopy may be required for biopsy or polyp removal [8]. Routine imaging investigations are often limited in detecting cancer due to its small size or the difficulty in delineating it from soft tissues. An appropriate treatment plan is based on a comprehensive clinical and paraclinical evaluation (combining the latest imaging techniques with tumor biomarkers and genetic analyses) [9].

In recent years, the quest for specific serum tumor markers has emerged as a critical aspect of tumor diagnosis [10]. The exploration of blood-based samples, particularly plasma, using Fourier transform infrared spectroscopy (FT-IR) represents a longstanding investigative approach [11]. Barlev et al. conducted a study utilizing infrared spectroscopy on peripheral blood mononuclear cells and plasma to facilitate early detection of colorectal cancer in a group of 62 individuals [12]. Generally, the studied samples by infrared spectroscopy collected from patients with CRC (and healthy volunteers) are peripheral blood mononuclear cells [12], blood plasma [12,13,14], blood serum, saliva, and colonoscopy fluids [8], and biopsy tissues [15,16,17,18,19] or structural disorders in CRC deoxyribonucleic acid (DNA) [20]. In addition, to FT-IR, Raman spectroscopy [21] was employed with much success, especially as a liquid biopsy technique [22]. More discussion related to FT-IR spectroscopy can be found in the Supplementary Information.

For human or animal studies, which usually involve a large number of subjects, statistical analyses of measured data are performed. There is a wide range of specialized software used for this purpose [23,24,25,26,27,28]. For more details, see the Supplementary Information.

Proton nuclear magnetic resonance (^1^H NMR) methods, including conventional 1D spectroscopy [29,30,31,32,33,34,35], J-resolved 2D spectroscopy, relaxation-edited, and diffusion-edited pulse sequences, are extensively employed for monitoring various metabolite groups [29]. The investigated biological tissues are diverse, such as urine [29,34], plasma [29] serum [29,30,32], sebum [31] and tissue extracts [29] like colorectal polyp [32], colon cancer samples [33], endometrium [35,36] or breast [37]. These sophisticated techniques are frequently coupled with statistical analyses, such as principal component analysis (PCA) [30,32,33,34,36], multivariate ROC curves (sensitivity versus 1-specificity) [32,33,34,37,38,39] or additional 2D NMR analyses to facilitate biomarker discovery, metabolic profiling, and other metabolomics applications. In recent years, artificial intelligence (AI) has emerged as a successful tool for addressing these challenges.

Blood plasma, collected from patients with colorectal cancer (CRC), either native or deproteinized, contains multiple structural and dynamic components. The structural components can be highlighted using FT-IR spectroscopy, while the dynamic components can be analyzed through ^1^H NMR relaxometry, specifically using 1D *T*_2_ distribution [14]. A previous study demonstrated [14] that these types of measurements could be used for CRC detection. This paper aims to investigate the sensitivity of 1D ^1^H NMR relaxometry and FT-IR spectroscopy to detect changes in CRC patients’ blood samples, interpreted as degree of healing (recovery after surgery that involves physical healing of the wound and restoration of bodily functions), at seven days after surgery by applying faster techniques compared to traditional histopathological methods [40], and to show how these changes can be used directly (specific for each patient), or by a further analysis which implies statistical analysis in principal components (PCA) in combination with ROC (and AUC) and predictions using a trained ANN based on machine learning algorithm for group analysis. Moreover, the utility of often-used blood plasma deproteinization will be discussed.

## 2. Materials and Methods

### 2.1. Volunteers and Patients

Informed consent was requested and obtained from all 20 volunteers and 10 patients prior to the clinical investigations. For this study, we used data from a group of 20 healthy volunteers aged between 26 and 65 years (mean age: 52) with no history of cancer and 10 colorectal cancer patients (both men and women), aged between 45 and 81 years (mean age 67.6). More information about the patients can be found in Table 1. This is a pilot study involving more than 40 patients with colorectal cancer, from which those 10 patients for whom preoperative and postoperative data are available were chosen.

### 2.2. Clinical Care After Surgery

The recovery period after surgery (removal of colon or rectal cancer-affected tissue) for the patients in this study ranged between 7 and 10 days, during which the CERAS protocol (ERAS protocols applied in colorectal surgery) was applied [41,42]. This protocol integrates 15 to 20 key variables that have been carefully selected to optimize each phase of the process and involves collaboration among a multidisciplinary team (surgeons, nutritionists and nurses) who strictly follow the supervisor’s instructions, including bed rest, mobility, hydration, and medication management. Patient monitoring included the evaluation of vital functions, periodic blood tests (monitoring of inflammatory markers, assessing liver function), and pain management (intravenous antiallergics [43] at scheduled times). On days 1 to 3, patients received an infusion diet. From day 4 onward, strained soup was gradually introduced within each patient’s tolerance level (days 4 to 6). On days 5 to 7, in addition to strained soup, soft foods such as dough-based items and light meat were incorporated.

With a high confidence one can declare that the major factor that influenced the 7 days period after surgery is the clinical intervention itself. Other factors, such as specific treatments, fluid intake, activity, diet or stress response are almost the same for all patients; therefore, one can expect to have approximately the same influence on the patient’s healing process. Other factors, such as treatments for specific health problems, age, genetics, stress, living conditions, etc., may affect the healing ability and are specific to each patient. The overall effect of all of these will be quantified by ^1^H NMR relaxometry and FT-IR spectroscopy.

### 2.3. Blood Plasma

For each participant, 6 milliliters of blood was drawn from a peripheral vein. The collected blood was then centrifuged at 2000 rpm for 30 min to separate its components. Afterward, the supernatant (plasma), was stored at −80 °C until analysis. Before FT-IR or NMR analysis, the samples were allowed to reach room temperature. To prepare deproteinized plasma samples, 100 µL of blood plasma was mixed with 900 µL of methyl alcohol, and centrifuged at 2000 rpm for 15 min. Subsequently, the deproteinized plasma was collected from the top of an Eppendorf tube. All sample types, including native and deproteinized blood plasma, were stored in a refrigerator until measurement. For more details, see Ref. [14].

### 2.4. ^1^H NMR Relaxometry

The ^1^H NMR relaxometry measurements were conducted using a low-field Bruker Minispec MQ 20 (Bruker Co., Ettlingen, Germany) spectrometer, at a frequency of 19.69 MHz [19,44,45,46]. The Carr–Purcell–Meiboom–Gill (CPMG) pulse sequence, as mentioned in Refs. [44,45,46,47,48,49], had two echo times: 70 µs and 500 µs. To fully measure the decay of liquid samples, 3000 echoes were recorded. The recycle delay (RD) was set to 3 s, and the measurements were accumulated over 64 scans. Data analysis was performed using a Laplace-like inversion algorithm [46,47,48,49], resulting in normalized transverse relaxation time *T*_2_ distributions, *f*(*T*_2_) [46,48,49]:

(1) $$M\left(\tau \right)=\int_{0}^{\infty }f\left(T_{2}\right)e^{-\frac{\tau }{T_{2}}}dT_{2}.$$

### 2.5. FT-IR Spectroscopy

For the FT-IR analysis, a Jasco 6200 FT-IR (Japan Spectroscopic Co., Ltd., Hachioji, Tokyo, Japan) spectrometer was used. To begin, 200 mg of KBr was ground using an agate mortar. The resulting powder was placed into a mold and compressed under a pressure of 15 metric tons to form a thin solid disc. Initially, a pure KBr disc was used as a background reference. Then, 40 µL of liquid sample was applied directly onto the surface of the KBr disc. The measurement range was set from 350 cm^−1^ to 4000 cm^−1^ with a resolution of 4 cm^−1^, and the FT-IR spectra were recorded over 64 scans, in approximately 1 min and 20 s, a time significantly lower than the drying time of the liquid sample.

## 3. Results

### 3.1. ^1^H NMR T_2_ Distributions

**Figure 1:**
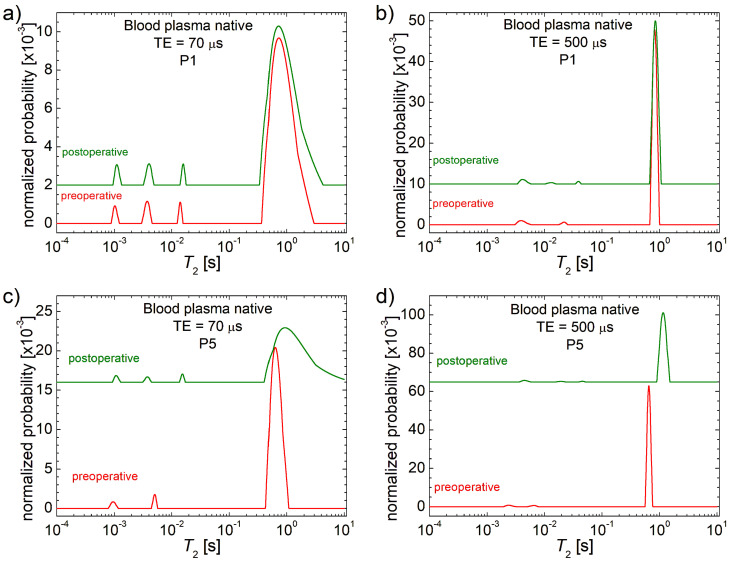
*T*_2_ distributions measured with two echo times TE = 70 μs and 500 μs for native blood plasma collected from patients (**a**,**b**) P1; (**c**,**d**) P5 with colorectal cancer preoperative (red) and postoperative at 7 days from surgery (olive).

The *T*_2_ distributions measured for native blood plasma collected from patients P1 and P5 with CRC shown as paired preoperative (red) and postoperative (olive, at 7 days after surgery) are presented in Figure 1. The *T*_2_ distributions measured for patients from P2 to P4 and from P6 to P10 are provided in Figures S1 and S2 from Supplementary Information. The measurements were performed using two echo times: TE = 70 µs (left), which highlights peaks at small relaxation times (*T*_2_), and TE = 500 µs (right), which enhances peaks at larger *T*_2_ values. For more details on the interpretation of *T*_2_ distributions, please refer to the Supplementary Information.

For the evaluation of surgical effects on the native and deproteinized blood plasma on soluble fraction concentration (main peak at large *T*_2_) and insoluble (small peaks at low-medium *T*_2_) fraction dynamics, we will simply compare the T_2_ distributions measured preoperatively and postoperatively for our 10 patients in the unsolvable fraction of native blood plasma. The most dramatic changes are observed in the evolution of patient P5. A substantial shift of the main peak towards larger *T*_2_ values suggests a decrease in soluble fraction concentration. Additionally, the appearance of a third peak in the insoluble fraction group indicates the presence of smaller-sized insoluble components.

**Figure 2:**
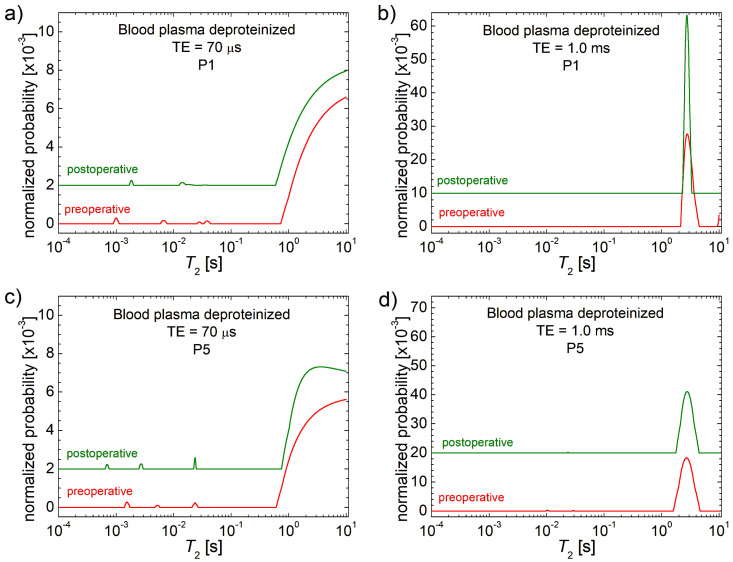
*T*_2_ distributions measured with two echo times TE = 70 μs and 1 ms for deproteinized blood plasma collected from patients (**a**,**b**) P1; (**c**,**d**) P5 with colorectal cancer preoperative (red) and postoperative at 7 days from surgery (olive).

The changes in *T*_2_ distributions measured for patients P2, P8 and P9 present no (TE = 70 µs) or small (TE = 500 µs) changes in the unsolvable fraction of native blood plasma (see also Figures S1 and S2 from Supplementary Materials). For patients P3 and P4, one can observe a small displacement of the most immobile unsolvable fraction after surgery to even the smallest values, indicating an increase in mass, while the peaks associated with the solvable fractions remain the same. For the rest of the patients, one can observe some changes to both solvable and unsolvable fractions of native blood plasma. Thus, patient P10 presents a shift of the major peak toward smaller *T*_2_ values, indicating an increase in solvable fractions after surgery, and no relative changes in the unsolvable fractions. Conversely, for patient P7, one can observe the same behavior for solvable fractions but also a change to unsolvable fractions where the most immobile fraction appears. For patient P6, one can observe a reverse behavior of solvable fractions in the sense that they are reduced in concentration (the main peak is shifted towards larger *T*_2_ values). And, at the same time, one can observe the apparition of a new peak located at approximately 200 ms (see Figure S1h). The same measurements were performed for deproteinized plasma. The comparative *T*_2_ distributions are presented in Figure 2 for patients P1 and P5, while the corresponding *T*_2_ distributions for P2 to P6 are presented in Figure S3 and in Figure S4 for P7 to P10. It can be observed that only the measurement performed at TE = 500 µs provides clarity for the main peak. In deproteinized plasma, methyl alcohol has a significant impact on *T*_2_ distributions leading to no major changes in soluble fraction peaks and considerably reducing the amplitude of insoluble fraction peaks.

### 3.2. FT-IR Spectra

**Figure 3:**
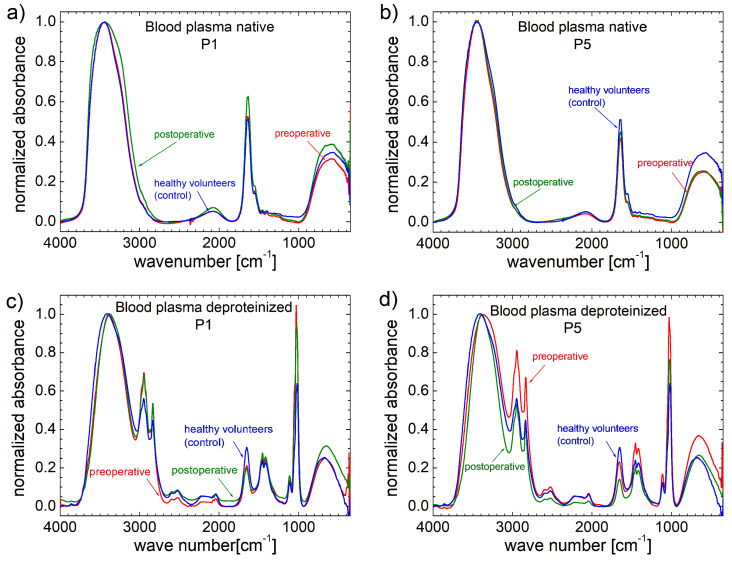
FT-IR spectra measured for native (top) and deproteinized (bottom) blood plasma collected from patients (**a**,**c**) P1 and from patient (**b**,**d**) P5 with colorectal cancer preoperative (red) and postoperative at 7 days from surgery (olive) compared with the average FT-IR spectra of 20 healthy volunteers (blue).

In the case of FT-IR spectra, the concentration of a specific component is directly proportional to the amplitude of the measured peak. The FT-IR spectra collected from 20 healthy volunteers were averaged (unlike in ^1^H NMR relaxometry—see the explanation in the dedicated technical section ^1^H NMR *T*_2_ distributions from Supplementary Information) and are presented as a blue line in Figure 3 for native blood plasma (on top) and for deproteinized plasma (on bottom). The FT-IR spectra measured for the patients P1 and P5 collected preoperatively (red) and postoperatively (olive) are comparatively shown in Figure 3. Compared to the FT-IR spectra of deproteinized plasma (Figure 3, bottom), those of native plasma are simpler (Figure 3, top). Five distinct spectral regions can be observed [8]: (i) a broad peak between ~350 and 1000 cm^−1^; (ii) a region with low absorbance between ~1000 and 1500 cm^−1^; (iii) a narrow peak with a right shoulder between ~1500 and 1800 cm^−1^; (iv) a broad peak with low amplitude between ~1850 and 2500 cm^−1^ and (v) a major peak between ~2700 and 3800 cm^−1^.

**Figure 4:**
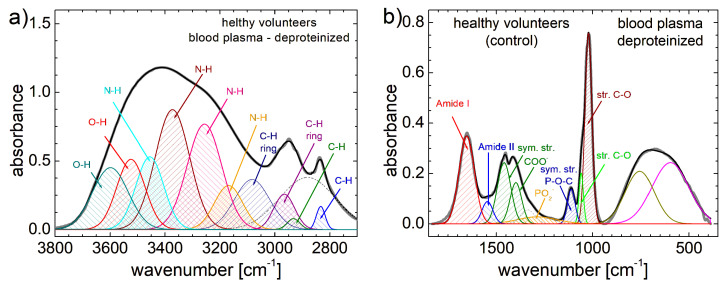

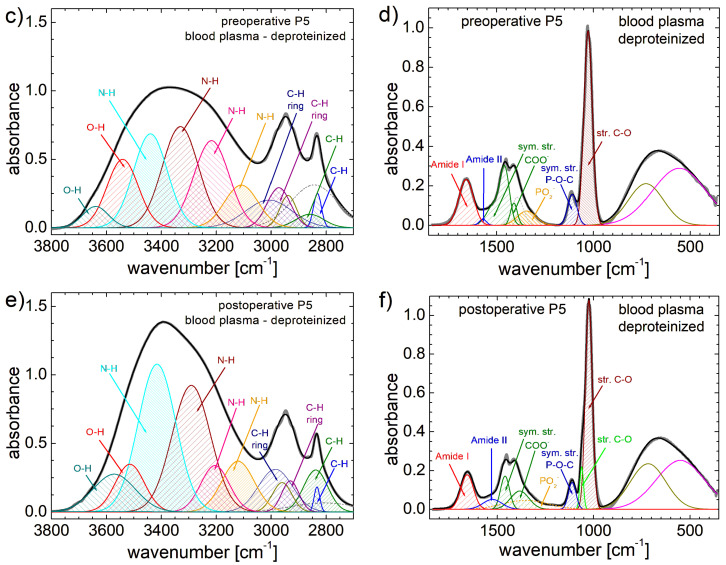
Deconvolution of FT-IR spectra (high wavenumber left column and low wavenumber right column) measured for deproteinized blood plasma collected from (**a**,**b**) healthy patients, and patient 5 with colorectal cancer preoperative (**c**,**d**) and postoperative at 7 days from surgery (**e**,**f**).

The decompositions of FT-IR spectra, measured for deproteinized blood plasma collected from a healthy volunteer and from patient P5 with CRC preoperatively and postoperatively, are presented in Figure 4. On the left column, the decomposition of FT-IR spectra plotted in the 2700–3800 cm^−1^ range (11 bands; 10 associated), while on the right column are the FT-IR spectra are shown in the 350–1850 cm^−1^ range (10 bands; 8 associated). A large number of components can contribute to the formation of the observed broad peaks. The FT-IR spectra measured for the other patients (see Figure S5), along with peak associations are extensively presented in Supplementary Information [13,14,50].

### 3.3. PCA Statistical Analysis and ROC Curves

Alternatively, to the time-consuming decomposition procedure, one can apply a statistical analysis of principal components (PCA). Such a PCA analysis was performed for the groups of FT-IR spectra recorded for native and deproteinized blood plasma and the primary plot (PC1 versus PC2) is presented. There is no clear clustering of results in three groups: healthy volunteers (represented with green triangles); patients with preoperative CRC (red squares); and patients with postoperative CRC (blue circles). For native plasma analysis on healthy volunteers, the following observations can be made: (i) one cluster formed by V1, V11 to V15 at negative PC1 (less than ~43.5) and above −4 for PC2; (ii) another cluster at negative PC2 (less than −34.8) and extended for PC1 from −21.7 up to 70.1; (iii) isolated V2, V3, V16 and V17. Moreover, there was not any differentiation (separate cluster formations in PCA) in data belonging to healthy volunteers based on their age (between young and old) observed.

**Figure 5:**
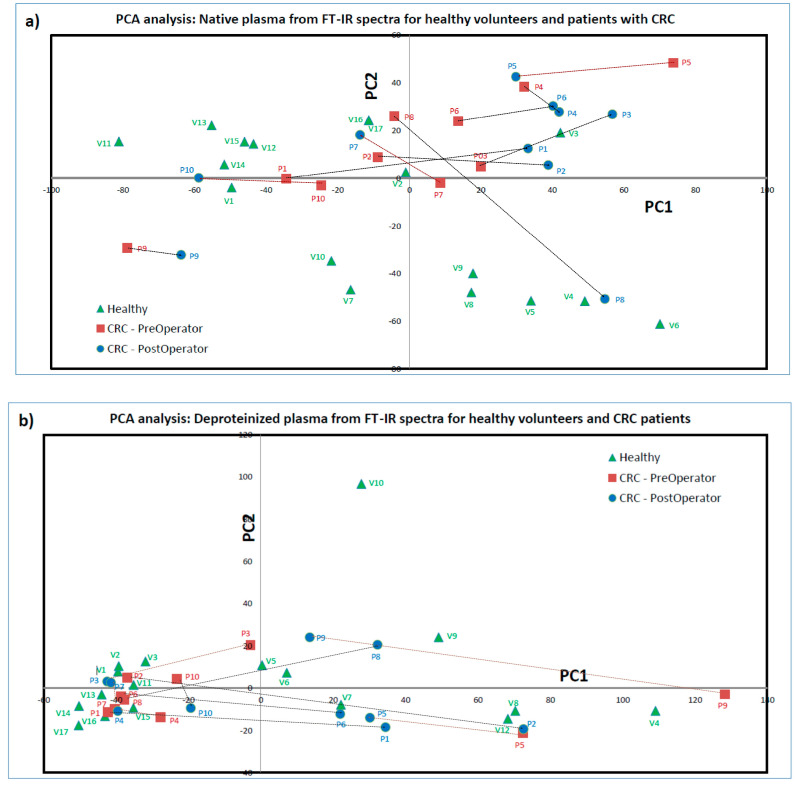
PCA analysis of FT-IR spectra measured for (**a**) native and (**b**) deproteinized blood plasma.

In Figure 5a, the evolution from preoperative to postoperative is marked with a dashed arrow for each patient. The arrow is black if the evolution goes from small PC1 values to large PC1 values and red if it goes in the opposite direction. The quantification of various behaviors from native blood plasma (Figure 5a) shows the following: (i) a small evolution like in the case of P9, which remains isolated at negative PC1 and PC2; (ii) an evolution towards the cluster of healthy clusters like in the case of P10 and P8; (iii) evolution towards a new state (at positive PC1 and PC2) represented by many postoperative individuals as in the case of P1, P2, P3, P4, P5 and P6; and (iv) small evolution towards into an incertitude area like in the case of P7, which (from the point of view of FT-IR spectra measured for native blood plasma), postoperatively, in the first approximation is similar to healthy volunteers V16 and V17 and in the second approximation is similar to P8 and P2 preoperatively.

Despite the fact that the FT-IR spectra recorded for deproteinized blood plasma (see Figure 3, bottom and Figure S6 in the Supplementary Information) appear more informative, the PCA analysis performed on these is not as clear as in the case of native blood plasma. One can see in Figure 5b that there is a large cluster of negative PC1 numbers formed by healthy volunteers in preoperative and postoperative cases. One can see another area at positive PC1 numbers where we only have two preoperative points belonging to the more discussed patient P5 and the above identified as isolated patient P9. The PC2 component does not seem to be able to induce a separation. In this analysis, one can observe several types of evolution: (i) small evolutions inside the nondiscriminatory area as in the case of P4, P7 and P10; (ii) medium evolutions as in the case of P3, which goes in the nondiscriminatory cluster or P5, which goes into an area populated with a representation of healthy volunteers and postoperative patients; and (iii) large evolutions as in the case of P2, P1, P6 and P8, which are going from nondiscriminatory cluster towards the healing healthy area, and P9, which goes from extremely right to the healing healthy area.

**Figure 6:**
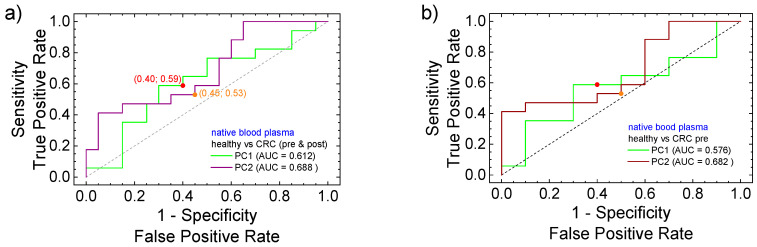

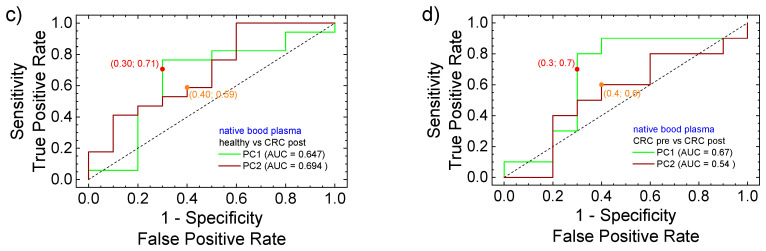
ROC curves calculated from PCA analysis data of FT-IR spectra measured for native blood samples for PC1 (green curve) and PC2 (brown curve) considering (**a**) healthy (positive) versus CRC (preoperative and postoperative); (**b**) healthy (positive) versus CRC (preoperative) (**c**) healthy (positive) versus CRC (postoperative) and (**d**) CRC (preoperative) (positive) versus CRC (postoperative). The optimal cutpoints are indicated for both parameters PC1 and PC2 as well as the area under the curve (AUC).

A better estimation of the PC1 and PC2 parameters to discriminate the measurements in the PCA analysis is provided by the associated receiver operating characteristic (ROC) curves on these parameters. The ROC curves (sensitivity versus 1, specificity) are presented in Figure 6 for native blood plasma and in Figure 6 for deproteinized blood plasma. Together with the ROC curves presented for the PC1 component (green) and the PC2 component (brown), the area under the curve (AUC) is also given, along with the optimal cut-off points. Four groups can be formed of healthy volunteers of patients with preoperative and postoperative CRC: (i) healthy (positive) versus CRC (preoperative and postoperative); (ii) healthy (positive) versus CRC (preoperative) (iii) healthy (positive) versus CRC (postoperative) and (iv) CRC (preoperative) (positive) versus CRC (postoperative). One can observe that the curves are close to the main diagonal, indicating that the groups are hard to separate.

The ROC curves show that the PCA analysis performed for native blood plasma separated the four formed groups much better compared to the PCA analyses performed on deproteinized blood plasma. This is observed from the corresponding AUC where PC1 for native blood plasma (see Figure 6a) was found to be between 0.576 (native versus CRC-pre) and 0.67 (CRC-pre versus CRC-post). The fact that PC1 (with the highest relevance) presents a high AUC comparing the native blood plasma collected from patients with preoperative and postoperative CRC indicates that a certain evolution after the surgery is observed (here, the cut-off point was located at 0.7 sensitivity and 0.3 specificity). The expectation was that the clearest separation to be observed between the group of healthy volunteers from the group of patients with preoperative CRC (with 1-specificity/sensitivity of 0.4/0.6). The largest AUC (0.694) was obtained for PC2 component in the evaluation of healthy volunteers versus postoperative CRC. This is a clear indication that the healing process is not oriented to a state described by healthily volunteers and, in this way supports, once more, the existence of a *post-operator state*. With the exception of the case where the analysis of native blood samples collected from pre- and postoperative CRC patients are compared, for the rest of the cases, the measured AUC for PC2 is larger than that measured for PC1. Then, compared to PCA, the PC2 separates the pairs of two from four groups better from the point of view of the ROC curves.

**Figure 7:**
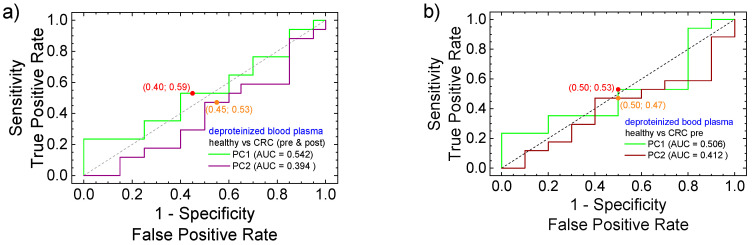

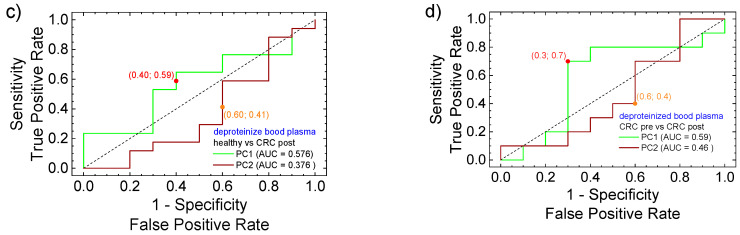
ROC curves calculated from PCA analysis data of FT-IR spectra measured for deproteinized blood samples for PC1 (green curve) and PC2 (brown curve) considering (**a**) healthy (positive) versus CRC (preoperative and postoperative); (**b**) healthy (positive) versus CRC (preoperative) (**c**) healthy (positive) versus CRC (postoperative) and (**d**) CRC (preoperative) (positive) versus CRC (postoperative). The optimal cutpoints are indicated for both parameters PC1 and PC2 as well as the area under the curve (AUC).

The areas under the curve (AUCs) calculated from ROC curved obtained from the PCA analysis of FT-IR spectra measured for deproteinized blood plasma are systematically lower compared to the corresponding AUCs measured for native blood plasma for the same four groups (see Figure 6 and Figure 7). Moreover, the AUCs calculated for PC1 are greater than 0.5 and all AUCs calculated for PC2 are smaller than 0.5, indicating that in that case, the true positive component was the partner. The largest AUC (0.59) was obtained for PC1 comparing the preoperative CRC group with the postoperative CRC group. This is an indication that a large the postoperative evolution exists. But, the fact that, in PC1, the AUC is smaller compared to the groups of healthy volunteers with the group of patients with postoperative CRC indicates that (according to FT-IR measurement on deproteinized blood plasma and PCA + ROC statistical analysis), postoperative at 7 days after surgery, the patients with CRC are closer to the healthy state than to the preoperative state. In conclusion, the values of AUC, with some exceptions, are greater than 0.5, indicating a majority of true positives compared to false positive; this suggests that the PCA analysis can discriminate (into a certain degree) between the studied groups. At the same time, since this discrimination may not be sufficient for a specialist practitioner, and another method of analysis (prediction) is required.

### 3.4. Machine Learning Prediction

**Figure 8:**
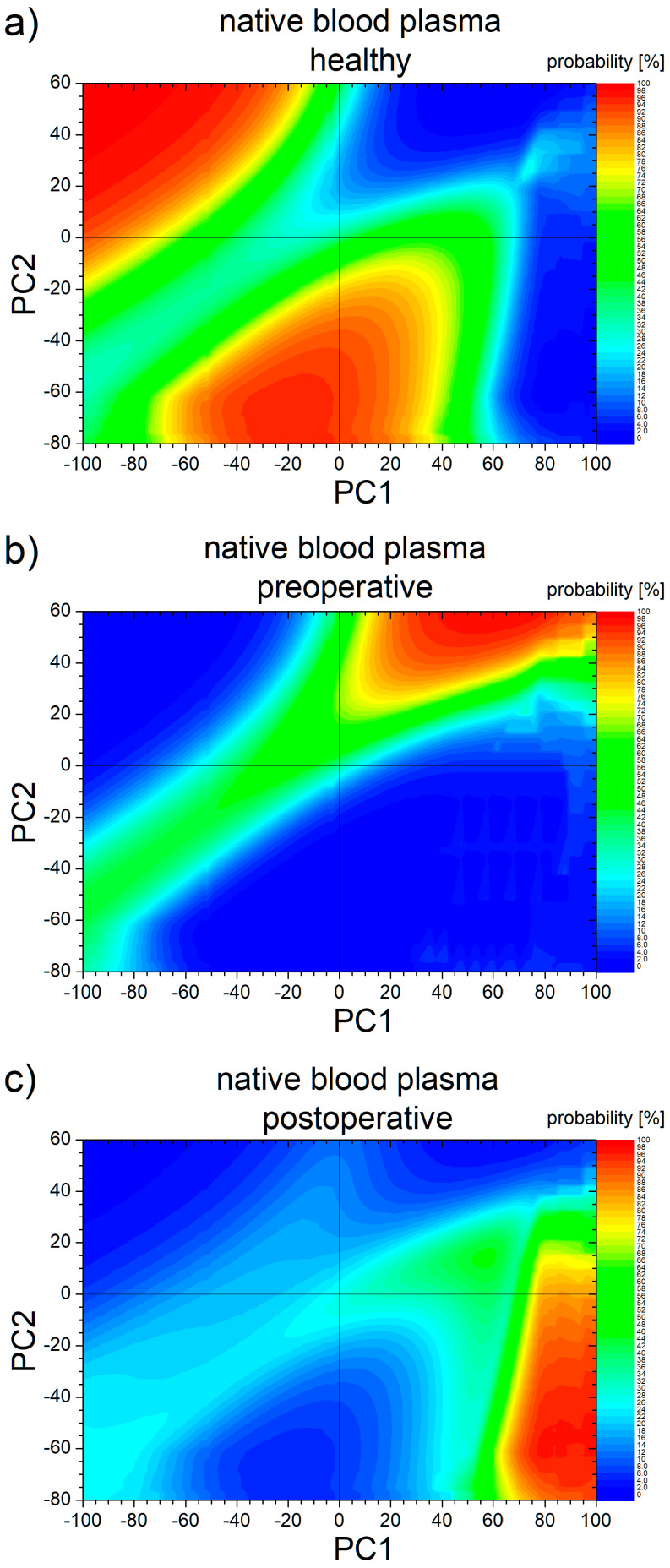
Machine learning used to predict the state of (**a**) health; and CRC probability (**b**) preoperative and (**c**) postoperative from 2D PCA analysis of FT-IR spectra measured for native blood plasma.

For patients diagnosed with CRC the state of healing after surgery is important to evaluated. In the present case, based on PCA analysis and predictions made by a machine learning algorithm, one can provide a probability for the state of health or of the healing degree. For prediction, we chose the 2D PCA data resulting from the analysis of native blood plasma. These data, together with the appropriate labels, constituted the input data on which the machine was trained. Then, each point from the PCA area was considered as a test-point for which the trained ANN predicted the probability for each state: healthy, preoperative CRC or postoperative CRC. The probability maps for all classes are presented in Figure 8. One can observe that points associated with healthy volunteers are located in two distinct areas (see Figure 8a) as: (i) at negative PC1 and positive PC2 values and (ii) at negative PC2 and around zero PC1 values (see the orange to red color). Medium probabilities (30–70%) are found to surround the areas with large probability (see the green color), and low probability is found at large PC1 values and at positive PC1 and PC2 values (blue-like color). One can notice an increased probability (~25%), compared with the surrounding points, and is found for values of about 70–80 for PC1 and 20–30 for PC2 due to the presence of V3. Our expectation was that at large PC1 values and negative PC2, a large probability of finding points belonging to healthy voluntaries is obtained. These data were probably *disturbed* by the presence of P8 postoperative in that area.

The probability distribution map for patients with CRC appears as anticipated: a quasi-diagonal, slightly shifted, elongated from negative PC1 and PC2 to positive PC1 and PC2 (see the green and red color in Figure 8b). What was unpredicted was just the exact probability associated with each point within these data. We expected a medium probability along the entire quasi-diagonal. The machine learning algorithm predicted an increased probability for positive PC1 values and large (>30) values of PC2.

Of particular interest was the predicted map associated with patient CRC at 7 days after surgery. In the PC1 vs. PC2 plot (see Figure 5a), the points associated with this category do not form a separate cluster. But these points are in the vicinity of points associated with one or two of the other categories. Surprisingly, one can find an isolated area (at large PC1 values and negative PC2 values) with a large probability (reddish colors in Figure 8c). In that area, only one point belonging to P8 is located postoperatively. Moreover, V6 is closer to that area than P8. For the rest of the probability map, one can find elevated probability (~40–70%—green-like colors) for positive PC1 values and positive (up to ~ 30) values for PC2 covering the area named healing.

## 4. Discussion

The proposed PCA analysis is not the ultimate analysis. For example, one could not observe a clear cluster representing the preoperative patients. The positions representing healthy volunteers are also spread. Nevertheless, for the PCA analysis performed for native plasma two *uncontaminated areas* were found. Only in a few cases is the pre to postoperative evolution directly toward areas *populated* by healthy volunteers. Thus, PCA analysis shows the existence of a *postoperative state*, away from the areas populated by representative values of preoperative patients, which we named the *healing area*. Many points corresponding to patients with postoperative CRC are either grouped, isolated or in close vicinity to points associated with healthy volunteers. In this sense, an extensive quantification of various behaviors is presented in the Supplementary Materials.

Comparing PCA analyses performed on both native and deproteinized blood plasma, one can observe similar behaviors, with clear a positive evolution for P08 (toward healthy area—native and healthy healing area—DP), P1, P2, P5 and P6 (towards *healthy healing* area). Patient P4, who preoperatively presented a close position to the healthy or healing healthy area after surgery shows a small positive evolution. Patient P7 also presents a small positive-like evolution. Patient P3 with a medium evolution also progresses toward the healing area of a nondiscriminatory cluster but is closer to the position occupied by healthy volunteers V1, V2 and V13. Patient P9 presents an unclear evolution as quantified from PCA of native plasma but progresses toward the healthy healing area according to PCA of deproteinized plasma. For patient P10, the PCA of native blood plasma shows an evolution toward healthy volunteers, and PCA on deproteinized blood plasma shows an unclear evolution.

The ROC curves show that the PCA on native blood plasma better separates the four groups compared to the PCA on deproteinized blood plasma. The fact that PC1 (highest relevance) presents a high AUC (cut-off point: 0.7 sensitivity and 0.3 specificity) when comparing patients with preoperative and postoperative CRC indicates a certain evolution after the surgery. From the point of view of ROC curves (a binary-group-oriented analysis), compared to PCA (a non-group-oriented analysis), the PC2 (second relevance in PCA) separates the pairs of two (from four) groups better. The AUCs calculated from the ROC analysis of deproteinized blood plasma are systematically lower compared to those measured for native blood plasma for the same four pairs (see Figure 6 and Figure 7).

PCA statistics, combined with ROC analysis to discriminate the healthy versus CRC from the FT-IR spectra, was performed earlier by Barlev et al., but they used a combined blood plasma with an entire biomolecular profile of peripheral blood mononuclear cells (PBMCs) with a validation AUC of 0.772 [12]. Tugrul et al. successfully used PCA (and LDA, SIMCA and HCA) multivariate data analyses on ATR–MIR spectroscopy, and also measured for blood plasma (and other bodily fluids) to recognize the changes in the spectral characteristics at a molecular level for CRC and healthy groups [13]. High-field NMR spectroscopy was used for excellent discrimination between healthy and colorectal cancer as presented by Gu et al. [32] from serum samples and by Kim et al. [34] from urine samples. Pacholczyk-Sienicka et al. [33] combined HF ^1^H MAS NMR spectroscopy, PCA and ROC curve analysis to discriminate between survivors and non-survivors of CRC in the context of specific metabolite ratio.

To our knowledge, there are no low-field ^1^H NMR relaxometry studies combined with FT-IR measurements, correlated with PCA statistics, ROC curve analysis and prediction maps using ANN for the evaluation of the healing process after surgery for patients with CRC. In a specific patient analysis (^1^H NMR *T*_2_ distributions), it was shown that P1–P4 and P8 present almost no change (pre- to postoperative), and P6 and P7 show a medium change. The largest changes were observed for patients P5 and P10. This result can be correlated with FT-IR-based PCA and one can observe (Figure 5a—native blood plasma) that the direction of evolution along PC1, from large to lower values (red line), is associated with patients P5, P10 and P7. For these three patients, the main peak (in *T*_2_ distributions) shifts from larger to lower *T*_2_ values, while for patient P6, the *T*_2_ shift occurs from lower to larger *T*_2_ values.

In all our analyses, patient P5 presents a positive evolution towards healing, a fact that is in total agreement with the clinical observation of the single patient for which the stage of diagnosis after surgery (0 in Table 1) indicated that it is healed. Contrary, patient P10, who also presented large changes in ^1^H NMR *T*_2_ distributions but in the opposite direction, is clinically classified with a stage of diagnosis after surgery of III, as with patient P6.

## 5. Conclusions

It has been proposed to use low-field ^1^H NMR relaxometry and FT-IR spectroscopy combined with PCA analysis, ROC and machine learning on native and deproteinized blood plasma to evaluate the state of healing in ten patients with colorectal cancer seven days after surgery. The ^1^H NMR *T*_2_ distributions are patient-oriented measurements and can indicate in each case if there is an evolution exists. FT-IR spectra can be directly used to evaluate the state of healing after surgery and can be compared to an average FT-IR spectrum associated with healthy volunteers. Unfortunately, the direct comparison of such FT-IR spectra leads to variations that are hard to clearly discuss in terms of healing. Fortunately, a statistical analysis such as PCA can be performed and a specific (patient-oriented) discussion can be had. Then, a group analysis (ROC statistics and AUC) was performed. Finally, the PCA data were used to train a machine learning-based ANN to predict the probability of healing. It was shown that it is possible to evaluate the state of healing using fast and inexpensive measurements based on affordable measurement instruments; the patients with CRC are closer to the healthy state than to the preoperative state; the native plasma can lead to better interpretation than the deproteinized blood plasma; and that the healing process is not linear (directly from preoperative to heaty as in a volunteer), but goes into an intermediate state that was named the *healing state*. From a clinical point of view, one can declare that the present study demonstrates a real potential application. The used methods are based on not-so-expensive equipment that requires non- (low field ^1^H NMR relaxometry) or inexpensive (KBr for FT-IR spectroscopy) reactants or consumables; is fast, taking 10–20 min per measurement (with sample preparation) compared with histopathological analysis; can provide multiple parameter values per (Fourier or Laplace) spectrum; presents a real potential of development with the new implementation of artificial intelligence; and can be improved in statistical analysis with each new analysis. Additionally, it works better for native blood samples, and the clinical time and cost for deproteinization are totally eliminated. From this perspective, a larger number of patients have to be investigated, forming more homogeneous study groups, and the proposed experimental methods correlated with statistical ones and artificial intelligence could explain better which factors are more relevant in the healing process.

## Figures and Tables

**Table 1:** Social and clinical parameters of the studied patients (P1 to P10).

Patient	P1	P2	P3	P4	P5	P6	P7	P8	P9	P10
Sex	F	M	M	M	M	M	F	M	F	M
Age (years)	45	67	66	67	81	67	72	81	55	75
Environment of Origin	urban	urban	rural	urban	urban	urban	urban	urban	urban	urban
ADK diagnoses	ML-R	L-R	S-C	LGM-C	L-R	MD-CRC	MD-R	CR	MD-C	WD-C
PET-CT/CT	PET-CT	CT	CT	CT	CT	CT	CT	CT	CT	CT
Neoadjuvant Treatment	RCT	RCT	RCT	NO	RCT	NO	RCT	NO	NO	NO
Smoking	no	yes	yes	no	no	yes	no	no	yes	yes
BMI categories	nw	uw	uw	uw	nw	nw	uw	nw	uw	uw
Histology	G2	G1	G2	G2	G2	G2	G2	G1	G2	G1
Stage of diagnosis after surgery	I	II	IV	III	0	III	I	I	III	III
Appetite Loss	yes	yes	yes	yes	no	yes	yes	no	yes	yes
Weight Loss	yes	yes	yes	yes	yes	yes	yes	yes	yes	yes
Fever	no	no	no	yes	no	no	no	no	no	no
Rectal Bleeding	yes	yes	no	no	yes	yes	yes	yes	yes	yes
Intestinal Transit Disorders	yes	yes	yes	yes	yes	yes	yes	yes	yes	yes
Drinking Alcohol	no	rarely	yes	no	rarely	rarely	no	rarely	rarely	rarely
Diabetes	dz type II	no	no	dz type II	no	dz type II	no	no	no	no
Anemia	no	no	yes	no	yes	no	no	no	yes	yes
Hemorrhoids	internal	no	no	no	internal	internal	Int. & ext.	internal	no	internal
Iron serum (µg/dL)	82	113	25	30	40	60	67	33	10	8
Hg preoperative (g/dL)	13.5	15.9	10.5	13.3	10.2	13.0	12.5	13.4	8.9	7.9
Hg 7 days (g/dL) postoperative	12.5	13.5	10.2	12.2	9.9	12.9	10.5	11.3	11.6	10.7
RDW-SD preoperative (fL)	49.9	45.8	48.4	53.2	46.8	45.4	48.3	54	79.3	44.1
RDW-SD at 7 days postoperative (fL)	47	43.1	53.8	51.1	45.6	45.5	47.3	53.2	89.9	66.4
Rayan score	1	3	-	-	0	-	3	-	-	-

ADK—Adenocarcinoma (MD-R—middle and lower rectal; L-R—Lower rectal; LGM-C—low-grade mucinous colonic; S-C—sigmoid colon; MD-CRC—moderately differentiated colorectal; MD-R—moderately differentiate rectal; CR—colorectal; MD-C—moderately differentiated colonic; WD-C—well-differentiated colonic); PET-CT—positron emission tomography-computed tomography; CT—computed tomography; RCT—radio-chemotherapy-treated; BMI—body mass index (uw—under-weight; nw—normal weight; ow—overweight; o—obese); histology categories (G1—well differentiated; G2—moderate differentiated; G3—poorly differentiated; S—signed cells; M—mucinous features); Hg—hemoglobin; RDW-SD—red cell distribution width—standard deviation.

## Data Availability

The original contributions presented in this study are included in the article and Supplementary Materials. Further inquiries can be directed to the corresponding authors.

## Supplementary Materials

The following supporting information can be downloaded at: https://www.mdpi.com/article/10.3390/cancers17050887/s1, **Sections:** CRC early detection; FT-IR and ^1^H NMR state of the art; Software used advanced analysis of measured data; ^1^H NMR *T*_2_ distributions; FT-IR spectra; The peaks association resulted from deconvolution of FT-IR spectra; The PCA analysis. **Figures:** Figure S1: *T*_2_ distributions measured with two echo times TE = 70 µs and 500 µs for native blood plasma collected from patients (a,b) P2; (c,d) P3; (e,f) P4; (g,h) P6 with colorectal cancer preoperative (red) and postoperative at 7 days from surgery (olive). Figure S2: *T*_2_ distributions measured with two echo times TE = 70 µs and 500 µs for native blood plasma collected from patients (a,b) P7; (c,d) P8; (e,f) P9; (g,h) P10 with colorectal cancer preoperative (red) and postoperative at 7 days from surgery (olive). Figure S3: *T*_2_ distributions measured with two echo times TE = 70 μs and 500 μs for deproteinized blood plasma collected from patients (a,b) P2; (c,d) P3; (e,f) P4; (g,h) P6 with colorectal cancer preoperative (red) and postoperative at 7 days from surgery (olive). Figure S4: *T*_2_ distributions measured with two echo times TE = 70 μs and 500 μs for deproteinized blood plasma collected from patients (a,b) P7; (c,d) P8; (e,f) P9; (g,h) P10 with colorectal cancer preoperative (red) and postoperative at 7 days from surgery (olive). Figure S5: FT-IR spectra measured for native blood plasma collected from patients (a) P2, (b) P3; (c) P4, (d) P6; (e) P7, (f) P8; (g) P9, (h) P10 with colorectal cancer preoperative (red) and postoperative at 7 days from surgery (olive) compared with the average FT-IR spectra of 20 healthy volunteers. Figure S6: FT-IR spectra measured for deproteinized blood plasma collected from patients (a) P2, (b) P3; (c) P4, (d) P6; (e) P7, (f) P8; (g) P9 and (h) P10 with colorectal cancer preoperative (red) and postoperative at 7 days from surgery (olive) compared with the average FT-IR spectra of 20 healthy volunteers. **References** [11,12,13,14,23,24,25,28,35,36,37,50] are cited in the supplementary materials.
