# Supplementary material for: Spectroscopic Nuclear Magnetic Resonance and Fourier Transform–Infrared Approach Used for the Evaluation of Healing After Surgical Interventions for Patients with Colorectal Cancer: A Pilot Study
Source: Cancers (Basel). 2025 Mar 5;17(5):887. doi: 10.3390/cancers17050887 (PMC11899188; doi:10.3390/cancers17050887)
Supplement: Supplementary file 1 [file cancers-17-00887-s001.zip › cancers-3466985-supplementary.pdf]

Supplementary Information

Spectroscopic Nuclear Magnetic Resonance and Fourier Transform – InfraRed approach used for the evaluation of healing after surgical interventions for patients with Colorectal Cancer

Lavinia Raluca Șaitiș[1,2], David Andras[3,4], Ioana-Alina Pop[5], Cătălin Șaitiș[6], Ramona Crainic[1,2] and Radu Fechete[2,*]

1 Babeş-Bolyai University, Faculty of Physics, Doctoral School, 1 Kogălniceanu, 400084, Cluj-Napoca, Romania; lavinia.dragan@ubbcluj.ro, ramona.crainic@ubbcluj.ro
2 Technical University of Cluj-Napoca, Faculty of Material and Environmental Engineering, 103-105 Muncii Bulevard, 400641, Cluj-Napoca, Romania; laviniadragann@yahoo.com , ramona.crainic95@gmail.com , rfechete@phys.utcluj.ro
3 County Emergency Hospital, Surgical Department, Clinicilor Str. 3-5, 400009, Cluj-Napoca, Romania; dr.andras@gmail.com
4 Iuliu Hatieganu University of Medicine and Pharmacy, Cluj-Napoca, Romania; andrasdavid88@elearn.umfcluj.ro
5 County Emergency Hospital, Radiology Department, Clinicilor Str. 3-5, 400009, Cluj-Napoca, Romania; ioanapop99@yahoo.com
6 Technical University of Cluj-Napoca, Faculty of Construction, 25 Baritiu, 400641, Cluj-Napoca, Romania; saitis@mail.utcluj.ro

*corresponding author: e-mail: rfechete@phys.utcluj.ro

CRC early detection

The early detection of CRC in symptomatic patients remains a persistent challenge. This intricate process commences when patients first recognize symptoms and extends through consultations with healthcare professionals, specialist referrals, and the subsequent wait for diagnostic procedures like colonoscopy. Among the general populace, lower abdominal symptoms are exceedingly prevalent and frequently prompt visits to healthcare providers. The issue lies in the often ambiguous and nonspecific nature of these symptoms, which demonstrate low sensitivity for CRC detection. Furthermore, the increasing demand for colonoscopies has emerged as a significant concern due to limited endoscopic resources, leading to delays in CRC diagnosis.[8]

FT-IR and ^{1}H NMR state of the art

Barlev et al. conducted a study utilizing infrared spectroscopy on peripheral blood mononuclear cells and plasma to facilitate early detection of colorectal cancer in a group of 62 individuals [12]. They meticulously recorded FT-IR spectra and meticulously identified distinct biomarkers, employing sophisticated analyses such as principal component and discriminant analyses. Their investigation highlighted a crucial spectral range within FT-IR, spanning from 1800 to 700 cm^{-1}, which encapsulates a wealth of biochemical data derived from blood plasma. Notably, significant changes were observed in specific bands: the 1080 cm^{-1} band, linked to symmetric phosphate vibrations; the 1400 cm^{-1} band, associated with symmetric stretching of COO^{-} from glutamate and amino acids such as aspartate; and symmetric CH_3 bending modes of protein methyl groups. These findings shed light on the potential utility of FT-IR spectroscopy as a promising tool for identifying early molecular alterations indicative of colorectal cancer [12]. This approach holds promise for advancing non-invasive diagnostic strategies and enhancing our ability to detect malignancies at earlier stages, ultimately improving patient outcomes and treatment efficacy. Recently Tugrul et al.

presents a biospectroscopic approach toward colorectal cancer diagnosis from bodily fluid samples (blood plasma, blood serum, saliva and colonoscopy disinfection/wash fluids) via attenuated total reflection–mid infrared (ATR–MIR) spectroscopy which was successfully combined with different multivariate data analysis such as principal component analysis (PCA), hierarchical cluster analysis (HCA), soft independent modeling by class analogy (SIMCA) and linear discriminant analysis (LDA) [13]. Their findings revealed significant changes in the concentrations of lipids, proteins, nucleic acids and carbohydrate biomolecules for cancer cases compared to the healthy-control cases. Moreover, an excellent classification for the studied groups were found for SIMCA and LDA analysis.

Assessing parameters like the tumor microenvironment (TME) and tumor budding (TB) is crucial in the diagnosis and prognosis of colorectal cancer (CRC) and its progression. Artificial intelligence (AI) has emerged in recent years as a successful tool for addressing these challenges. *In vivo* study involving NMR measurements using a 3 T magnetic field human tomograph of endometrial cancer [35, 36] and using a 1.5 T magnetic field human tomograph of breast cancer [37] were also reported. Additional to NMR spectroscopy, authors report ^{1}H NMR relaxometry measurements, i.e. a diversity of parameter maps of cancerous tissues such as T_2-maps (endometrial [36] and breast [37]), T_1-maps and spin density ($\rho_{^1H}$) maps [36]. The application of low-field NMR relaxometry for studying blood plasma of patients with CRC is underexplored in existing literature [14].

Software used advanced analysis of measured data

SEERStat (version 8.3.6) was utilized to calculate for example the age-adjusted colorectal cancer (CRC) incidence and mortality rates, standardized to the 2000 US population across 19 age groups, and expressed per 100,000 individuals [23]. Some

results are largely discussed by Siegel et al. [24]. For example, the rate ratios were determined along with 95% confidence intervals (95% CIs). Incidence trends were derived from rates adjusted for delays in reporting using race-specific and age-specific delay factors provided by US Cancer Statistics [25]. This adjustment for reporting delays accounts for the additional time required for the comprehensive registration of cases, thereby providing a more accurate reflection of recent cancer trends [26]. Incidence and mortality trends were quantified using Joinpoint regression analysis (version 4.7.0.0; NCI) [24]. The lifetime risk of developing cancer was calculated using the NCI's DevCan software (version 6.7.7) [28]. Other software can fulfil the same task (e.g. Origin®, MathLab®, MathCad®, Mathematica®, etc) usually are used by experimented scientist.

^{1}H NMR T_2-distributions

For any person, important information is to find out a clinical diagnosis of health or illness. Thus, for the evaluation of effects of surgery on the native and deproteinized blood plasma on solvable fraction concentration and unsolvable fraction dynamics we propose to simply compare the changes in the T_2-distributions measured for our patients preoperative and postoperative. One cannot compare each distribution with an *average of T_2-distributions measured for a group of healthy patients* since such T_2-distributions simply cannot be calculated. Here (contrary to FT-IR spectra – which will be discussed later), the average procedure, if applied, should assume mediation along the horizontal axis (the T_2-axis) and will not imply that, for a given T_2-value, should be calculated as averaged intensity.

The T_2-distributions measured for native blood plasma collected from patients with colorectal cancer presented as a pair of preoperative (red) and postoperative at 7 days from surgery (olive) are presented in Fig. S1 for P2 to P6 and in Fig. S2 for P7 to P10. The measurements were performed with two echo times (TE = 70 μs – left and TE

= 500 μs – right) to observe the peaks appearing at small relaxation time T_2 (small TE) and to clarify the peaks appearing at large T_2-values (large TE). A small echo time, TE will artificially broaden the peaks appearing at large T_2-values, while a large TE will filter the fast-decaying components (small T_2-values). As a general remark one can observe four peaks: i) three of them located at T_2-values below 20 ms (for TE = 0.07 ms) and ii) one, the main peak, located at T_2-values larger than 600 ms (see the right figures with TE = 0.5 ms). From left to right (i.e. from lower to larger T_2-values) the peaks describe components of native blood plasma (containing ^1H) with increased mobility. Then we will refer to these components as rigid, semi-rigid, intermediate and mobile. As expected, the majority of ^1H is located in the most mobile components, most probably the water from blood plasma. Distilled water presents a peak, in the T_2-distribution located at 3 s. If, in the water some dissolved fractions are present, then the corresponding peak is shifted towards lower T_2-values. This shift is proportional with the concentration of dissolved fraction but is also sensitive to the type of fraction (having magnetic properties, such as paramagnetic particles). Usually, the unsolvable fractions can be observed as peaks appearing at much smaller T_2-values (in our case one can see three of them). In such cases, ^1H may originate directly from the unsolvable fractions (especially if the sample is biological), which is associated to a strong molecular restriction (rigid component) and are characterized by small T_2-values (typically under 1 ms). Indirectly, ^1H with reduced mobility can originates from solvent, or more specific for our case, from water molecules attached to unsolvable fractions having different dimensions. Those which are attached to larger molecules present a reduced mobility (the rigid or semi-rigid components) and those which are attached to smaller fraction present a larger mobility (the intermediate components).

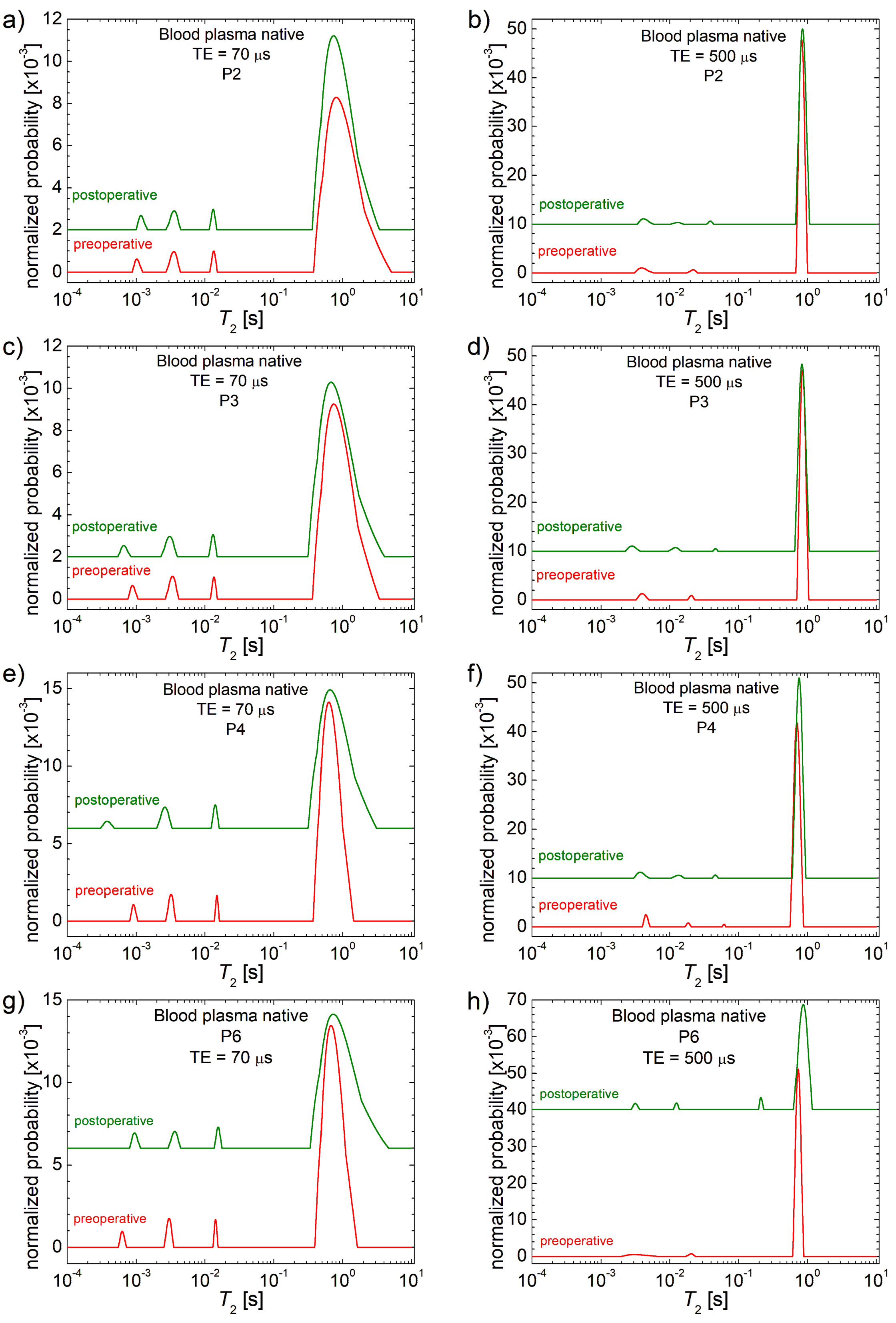

Figure S1 T_2-distributions measured with two echo times TE = 70 μs and 500 μs for native blood plasma collected from patients a) and b) P2; c) and d) P3; e) and f) P4; g) and h) P6 with colorectal cancer preoperative (red) and postoperative at 7 days from surgery (olive).

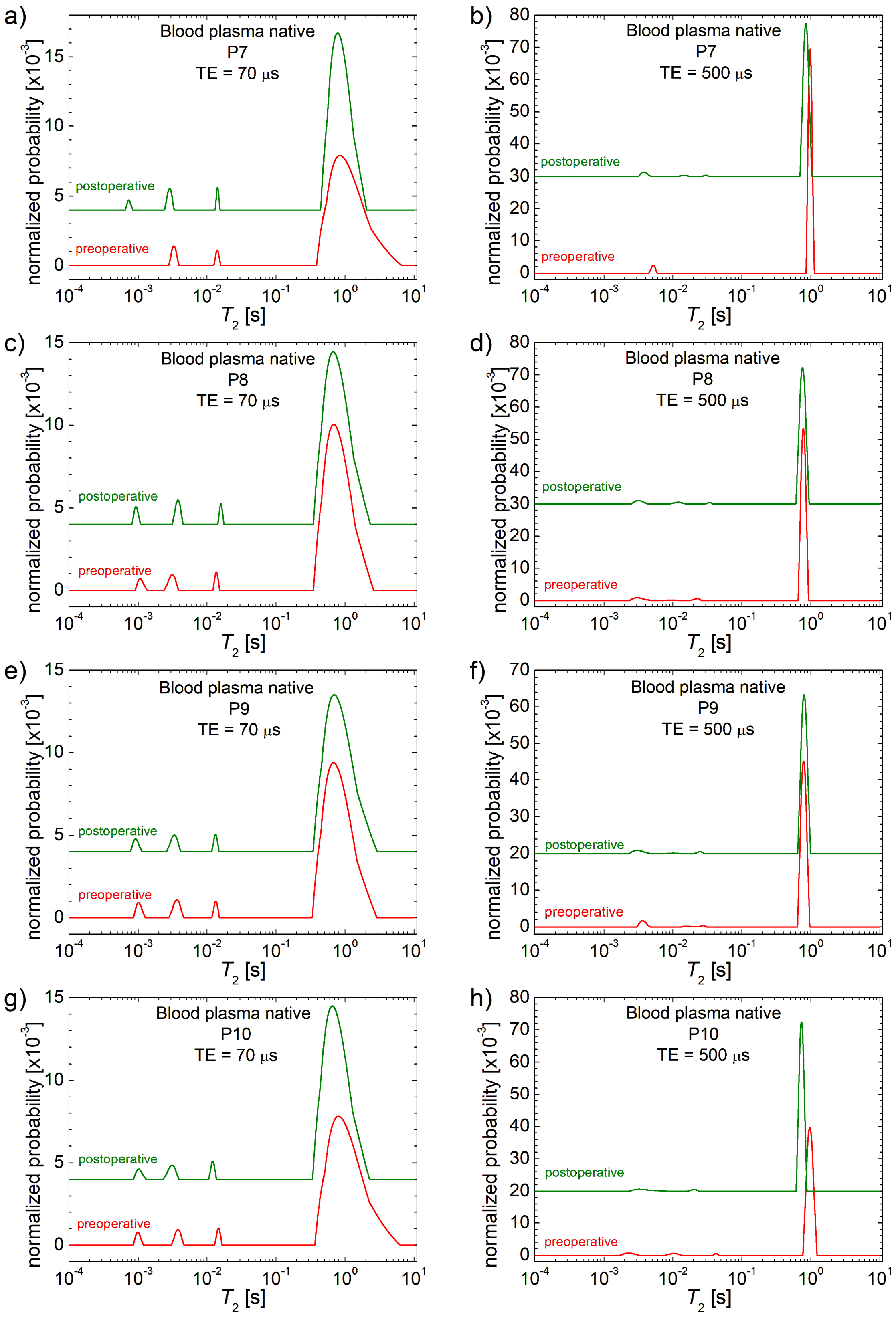

Figure S2 T_2-distributions measured with two echo times TE = 70 μs and 500 μs for native blood plasma collected from patients a) and b) P7; c) and d) P8; e) and f) P9; g) and h) P10 with colorectal cancer preoperative (red) and postoperative at 7 days from surgery (olive).

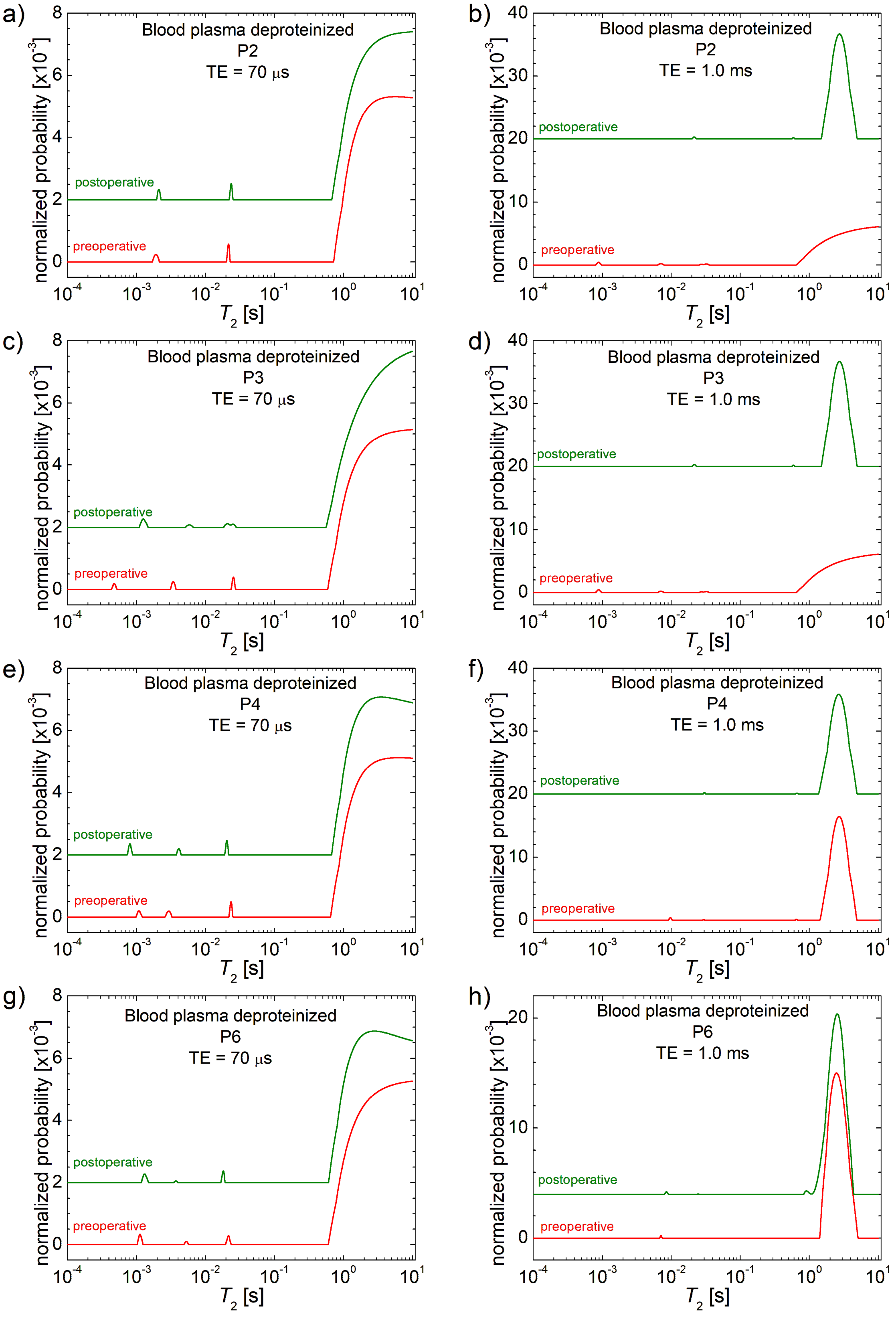

Figure S3 T_2-distributions measured with two echo times TE = 70 μs and 500 μs for deproteinized blood plasma collected from patients a) and b) P2; c) and d) P3; e) and f) P4; g) and h) P6 with colorectal cancer preoperative (red) and postoperative at 7 days from surgery (olive).

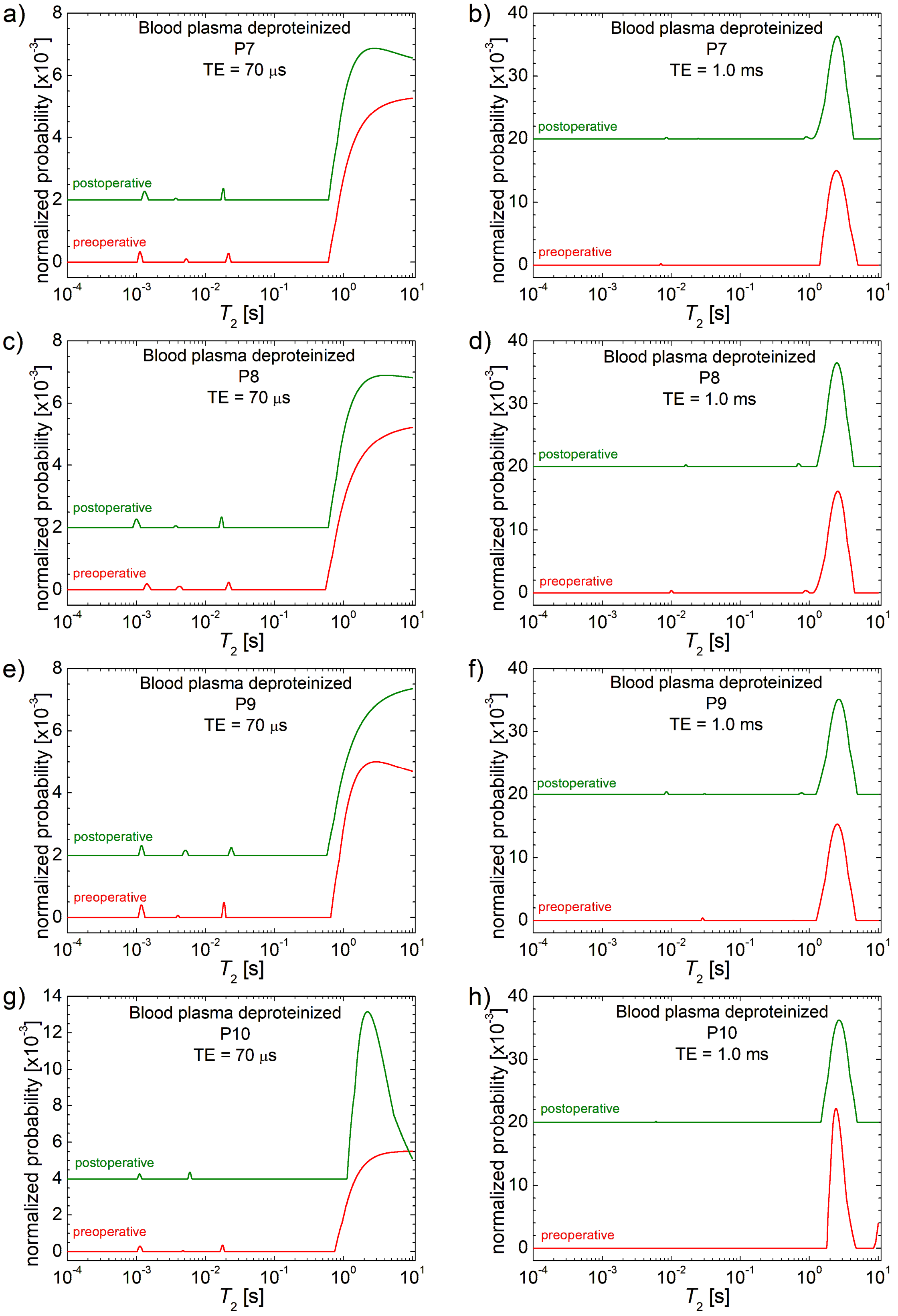

Figure S4 T_2-distributions measured with two echo times TE = 70 μs and 500 μs for deproteinized blood plasma collected from patients a) and b) P7; c) and d) P8; e) and f) P9; g) and h) P10 with colorectal cancer preoperative (red) and postoperative at 7 days from surgery (olive).

FT-IR spectra

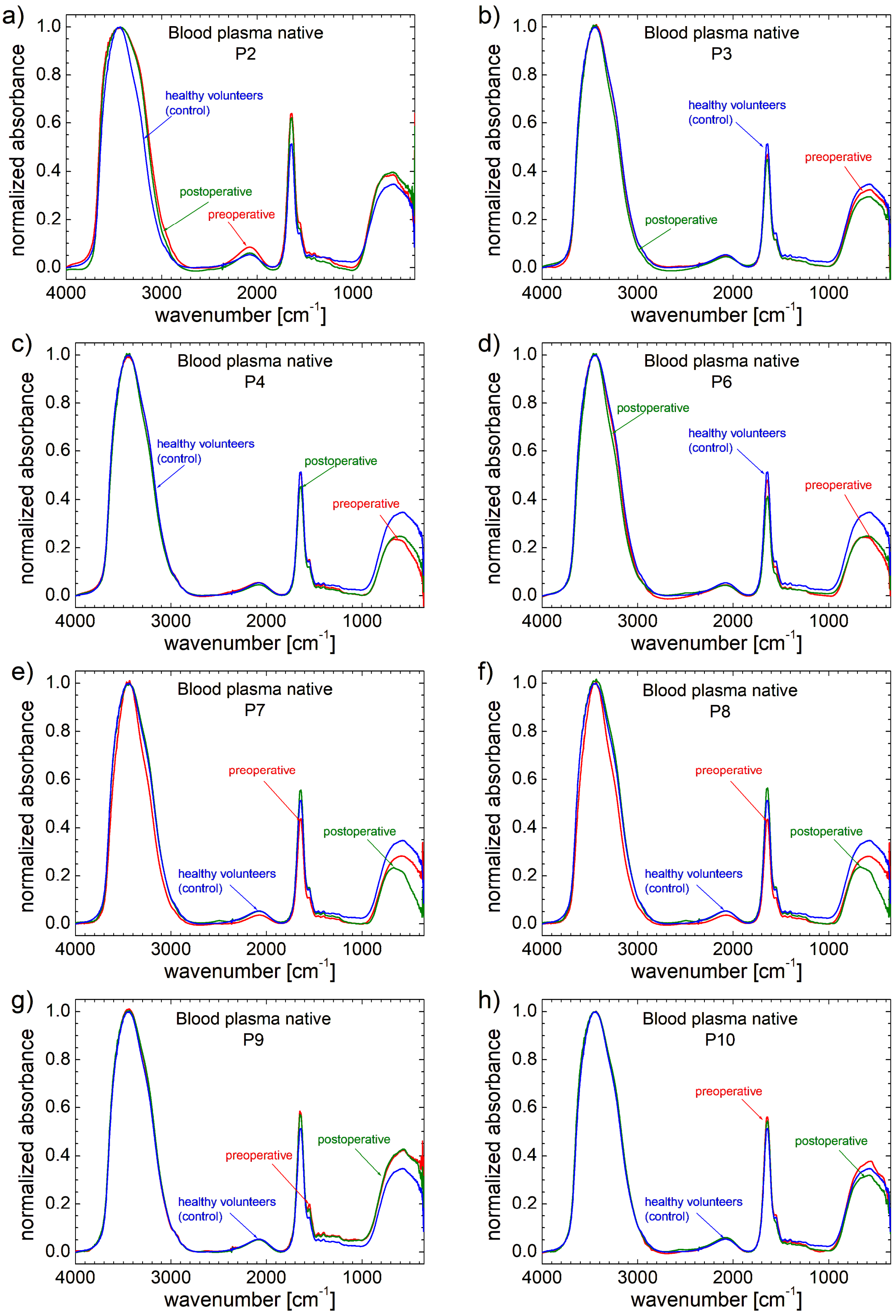

Figure S5 FT-IR spectra measured for native blood plasma collected from patients a) P2, b) P3; c) P4, d) P6; e) P7, f) P8; g) P9, h) P10 with colorectal cancer preoperative (red) and postoperative at 7 days from surgery (olive) compared with the average FT-IR spectra of 20 healthy volunteers.

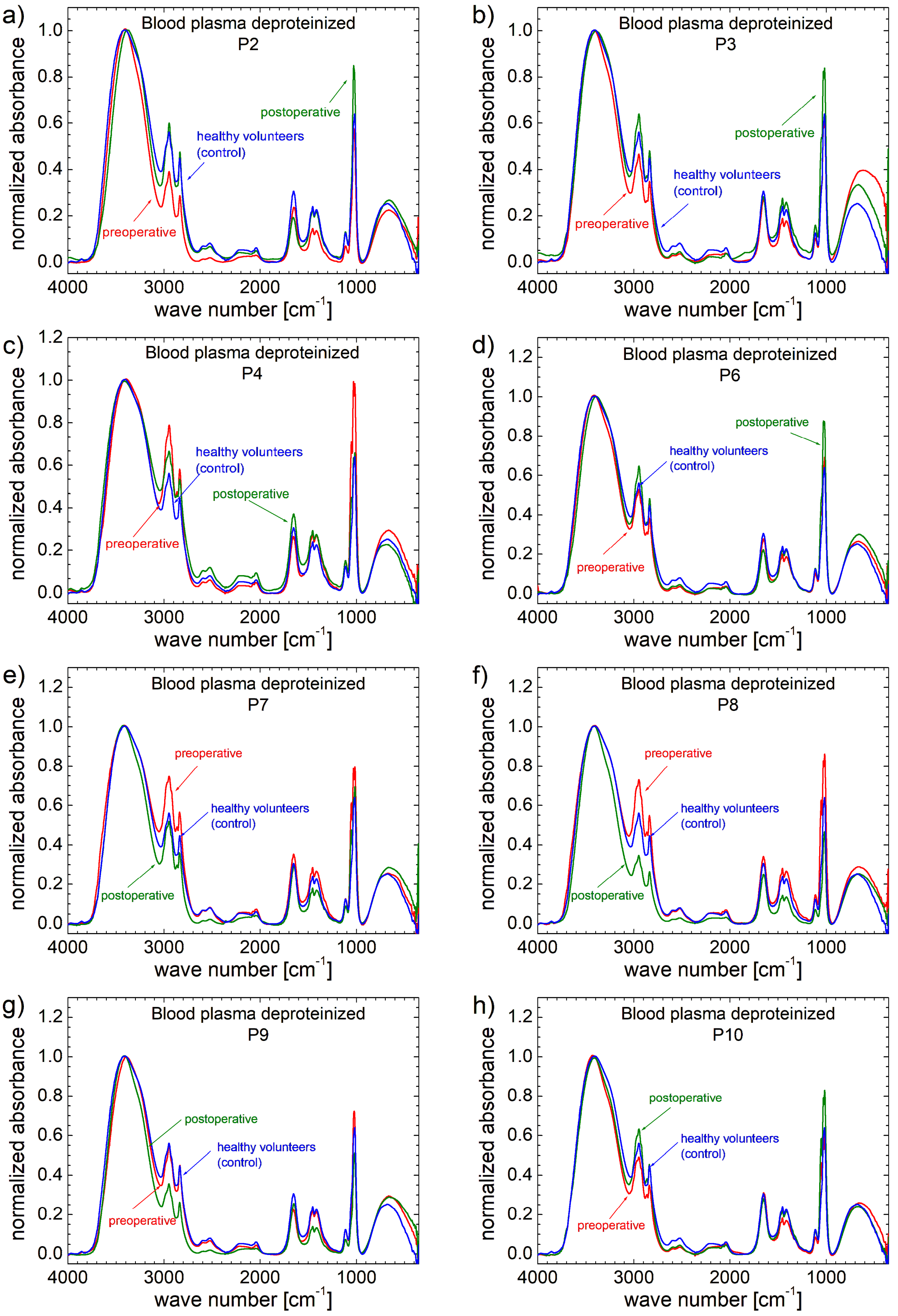

Figure S6 FT-IR spectra measured for deproteinized blood plasma collected from patients a) P2, b) P3; c) P4, d) P6; e) P7, f) P8; g) P9 and h) P10 with colorectal cancer preoperative (red) and postoperative at 7 days from surgery (olive) compared with the average FT-IR spectra of 20 healthy volunteers.

The peaks association resulted from deconvolution of FT-IR spectra

In the range of large wavenumbers one can observe (see Figure 4 from main document) that the broad peak extended from app. 2900 cm^{-1} up to 3800 cm^{-1} consist of two peaks associated to O-H bonding [50] as follows: i) first one located at ~3600 cm^{-1} (HV – healthy volunteer) displaced to ~3637 cm^{-1} for P5 preoperative and moved to ~3572 cm^{-1} for P5 postoperative and ii) second one located at ~3525 cm^{-1} for HV displaced to ~3540 cm^{-1} for P5 preoperative and moved to ~3515 cm^{-1} for P5 postoperative. Another four peaks are associated to N-H bonding[13, 14, 50] as follows: i) the first one located at ~3456 cm^{-1} for HV was displaced to ~3439 cm^{-1} for P5 preoperative and moved to ~3415 cm^{-1} for P5 postoperative; ii) the second one located at ~3373 cm^{-1} for HV was displaced to ~3330 cm^{-1} for P5 preoperative and moved to ~3290 cm^{-1} for P5 postoperative; iii) the third one located at ~3256 cm^{-1} (peptidehydroxyl groups [13]) for HV was displaced to ~3217 cm^{-1} for P5 preoperative and moved to ~3203 cm^{-1} for P5 postoperative and iv) the fort one located at ~3167 cm^{-1} for HV was displaced to ~3108 cm^{-1} for P5 preoperative and moved to ~3119 cm^{-1} for P5 postoperative. One can observe also two peaks usually associated to the C-H-ring [14, 50] as follows: i) the first one located at ~3081 cm^{-1} for HV was displaced to ~3006 cm^{-1} for P5 preoperative and moved to ~2983 cm^{-1} for P5 postoperative; ii) the second one located at ~2965 cm^{-1} for HV was displaced to ~2973 cm^{-1} for P5 preoperative and moved to ~2961 cm^{-1} for P5 postoperative. The last set of two peaks is associated to C-H bonding [14, 50] as follows: i) the first one located at ~2931 cm^{-1} for HV was displaced to ~2858 cm^{-1} for P5 preoperative and moved to ~2837 cm^{-1} for P5 postoperative; ii) the second one located at ~2833 cm^{-1} for HV was not displaced and it is located ~2932 cm^{-1} for P5 preoperative and not moved since remains at ~2933 cm^{-1} for P5 postoperative.

In the region corresponding to molecular back bonding (small wavenumber) on can find the peak associated to Amide I [13, 14, 50] located at ~1653 cm^{-1} for HV is

slightly displaced to ~1657 cm^{-1} for P5 preoperative and moved back to ~1655 cm^{-1} for P5 postoperative. The peak associated to Amide II [13, 14, 50] located at ~1546 cm^{-1} for HV is displaced to ~1568 cm^{-1} for P5 preoperative and moved back to ~1522 cm^{-1} for P5 postoperative. Another doublet that can be found in the FT-IR spectra in this region is associated to symmetrically stretching of COO^- functional groups [13, 14, 50]. These can be located at ~1461 and 1398 cm^{-1} for HV and are slightly displaced to ~1457 and 1411 cm^{-1} for P5 preoperative and moved back for ~1460 and pushed forward for 1383 cm^{-1} in the FT-IR spectra measured for P5 postoperative. A broad peak is represented by the vibrations due to PO_2^- functional group [13, 14, 50] which can be found at ~1287 cm^{-1} for HV but is narrower and displaced to ~1346 cm^{-1} for P5 preoperative and broader again but moved away to ~1353 cm^{-1} for P5 postoperative. The symmetrically stretching of P-O-C functional group [13, 14, 50] appears as a relatively small and relative narrow peak located at ~1110 cm^{-1} for HV remaining at ~1109 cm^{-1} for P5 preoperative and moved back to ~1110 cm^{-1} for postoperative. One can say that the position of this functional group in FT-IR spectra is less sensitive to the state of the subject (healthy volunteer or patient with colorectal cancer pre- or post-operative). The most intense peak is associated to C-O chemical bond vibrations [13, 14, 50]. A slight shoulder appear at large wavenumbers therefore the full peak was considered to be composed from two peaks a small and narrow one and a dominant one, which may also be considered narrow. These can be located at ~1059 and 1021 cm^{-1} for HV and are merging to a single peak located ~1026 cm^{-1} for P5 preoperative and separated again in two peaks located at ~1061 cm^{-1} and 1024 cm^{-1} in the FT-IR spectra measured for P5 postoperative. The FT-IR spectra contain also two broad peaks at small wave numbers which are not assigned.

The PCA analysis

The simple procedure of PCA analysis can be applied only on the FT-IR spectrum and not on the T_2-distributions. The reason is that the x-axis of a spectrum (Fourier for FT-IR and Laplace for T_2-distributions) becomes the parameters of PCA analysis. In FT-IR spectroscopy the wavenumber correlated with vibrations of whole or a part of molecules can be directly associated with specific functional groups (e.g. Amide I, amide II) and from sample to sample presents small deviations. With other words the absorbance of a sample component is seen as a peak characterized by position, width and integral area. The variations of peaks' positions for the studied samples are smaller compared with the distances between peaks. Therefore, the peak's association in FT-IR spectroscopy, usually, can be made with high accuracy. Conversely, in Laplace spectroscopy (the NMR T_2-distributions) the peaks may change dramatically the position (more than the peaks' width) and will no longer belonging to the same group, therefore the T_2-values can't be used as parameters for PCA analysis.

Reading the loadings of PC1 and PC2 data (not presented here) for PCA analysis performed on native blood plasma (Fig. 5a in the main document) one can say that 90 % of the influence on PC1 are the FT-IR spectroscopic absorbance between: i) ~1500 and ~1750 cm^{-1} (Amide I and Amide II), ii) ~2000 and ~2300 cm^{-1} (which usually is cut-of [13] or observed as a small broad band [11]) and iii) between 2900 and ~3700 cm^{-1} (O-H, N-H and C-H ring [13, 14, 50]). In the same time 90 % of the influence on PC2 are the FT-IR spectroscopic absorbance found between: i) ~900 cm^{-1} and ~1300 cm^{-1} (C-O stretching, symmetrically stretching of P-O-C bonds [13, 14, 50]), ii) ~2200 and ~2450 cm^{-1} (a combination of hindered rotation and O-H bending (water) [50]) and iii) between 3700 and ~4000 cm^{-1} (O-H vibrations [13, 14, 50]). In PC1, the position in FT-IR spectra with influence greater than 90 % is found in the ranges of: i) ~500 and ~850 cm^{-1}; ii) ~1000 to 1050 and ~1080 to 2020 cm^{-1} (belonging to C-O stretching); iii) ~1350 to ~1600 cm^{-1} (to PO_2^-, symmetrically stretching of P-O-C, symmetrically

stretching of COO^- functional groups and Amide II); iv) ~2000 to ~2300 cm^{-1} (many times not observed or observed as a combination of hindered rotation and O-H bending (water) [50]); and from v) ~2400 to ~3700 cm^{-1}. Basically, PC1 was influenced by the entire relevant FT-IR spectrum.
